# Heat‐to‐Electricity Conversion Using Barium Strontium Titanate Multilayer Capacitors

**DOI:** 10.1002/advs.77106

**Published:** 2026-08-21

**Authors:** Fan Ni, Junning Li, Uros Prah, Youri Nouchokgwe, Yves Fleming, Ashwath Aravindhan, Moiz Khalil, Torsten Granzow, Sakyo Hirose, Veronika Kovacova, Emmanuel Defay

**Affiliations:** ^1^ Luxembourg Institute of Science and Technology (LIST) Esch‐sur‐Alzette Luxembourg; ^2^ Department of Physics and Materials Science University of Luxembourg Esch‐sur‐Alzette Luxembourg; ^3^ Murata Manufacturing Co. Nagaokakyo Kyoto Japan

**Keywords:** energy harvesting, lead‐free ceramics, Olsen cycle, pyroelectric effect

## Abstract

Pyroelectric materials are attractive for converting heat into electricity, yet the best performance is obtained with lead‐containing ceramics, remaining a major environmental concern. Here, we address this problem by developing lead‐free barium strontium titanate multilayer capacitors (Ba_0.65_Sr_0.35_Ti_0.998_Mn_0.002_O_3_ MLCs). A maximum energy density of 3.7 J cm^−3^ is achieved under an applied field of 300 kV cm^−1^ across a 170 K temperature span in an Olsen electro‐thermodynamic cycle. We conducted Olsen cycles with two approaches: electric displacement‐electric field (*D‐E*) loops and stepwise control of temperatures and voltages, which provide consistent results. In addition, we found that Ba_0.65_Sr_0.35_Ti_0.998_Mn_0.002_O_3_ MLCs show a second‐order ferroelectric–to‐paraelectric (FE‐to‐PE) phase transition, which shifts to higher temperatures by the applied electric field. This provides a broad and practical temperature window for low‐grade waste heat recovery. Our study shows that the performance of pyroelectric energy harvesting in barium strontium titanate materials is comparable to their conventional lead‐based counterpart (3.6 J cm^−3^ in lead scandium tantalate in similar conditions), revealing the promising prospects of sustainable energy recovery applications.

## Graphical abstract

Lead‐free barium strontium titanate multilayer capacitors convert waste heat into electricity via the Olsen electro‐thermodynamic cycle. These capacitors achieve an energy density of 3.7 J cm^−3^, comparable to the performance of lead‐based ceramics. Their high dielectric strength and broad operating temperature window below 100°C make them practical for low‐grade waste heat recovery. This work offers a sustainable path toward energy harvesting.

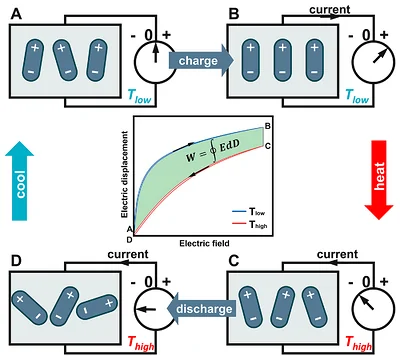

## 1 Introduction

Electricity is emerging as a key contributor to meeting energy service demand in every scenario [1]. A typical example is the rapid advancement and widespread application of artificial intelligence technologies, making the need for a sustainable power supply more urgent than ever. At present, global electricity production is dominated by thermal‐to‐electric conversion [2], yet a large share of input energy is rejected as waste heat [3]. Waste heat is generally classified into three categories: low temperature (below 100°C), medium temperature (100°C–300°C), and high temperature (above 300°C) [3]. Low‐temperature waste heat, often referred to as low‐grade, is typically overlooked due to its few end uses, low conversion efficiency, and the difficulty of recapturing it from diffuse sources [4]. However, its potential is significant because over 60% of total waste heat is low‐grade [3], making it an attractive target for recovery.

Thermoelectric and pyroelectric materials are solid‐state generators that directly convert heat into electricity. Thermoelectric materials use temperature gradients to output energy with the Seebeck effect [5]. These materials require maintaining temperature differences in the material system as substantial as the external ones. Thus, a huge surface area for heat exchange is necessary to improve the output work [6]. Besides, thermoelectric materials typically have high heat conductivity, which reduces the achievable temperature gradients [7]. These constraints result in bulky systems with low efficiency [8] and impede low‐grade heat harvesting below 100°C. Alternatively, pyroelectric materials can directly convert temporal temperature changes into electricity, offering a viable approach to utilize this untapped resource.

Recent studies have demonstrated that pyroelectric lead scandium tantalate (PST) materials show promise for efficiently converting thermal energy into electricity [9, 10, 11]. However, toxicity and high cost limit its applicability, and challenges remain in sustainable energy technologies. Alternative ferroelectric materials face challenges. For example, polymers based on polyvinylidene fluoride (PVDF) suffer from large leakage current and dielectric losses [12, 13]. Lead‐free materials like K_0.5_Na_0.5_NbO_3_ (KNN)‐based [14] and bismuth layer‐structured ferroelectrics (BLSF) ceramics [15] have relatively limited pyroelectric performance.

Among lead‐free pyroelectric materials, barium titanate (BaTiO_3_)‐based ferroelectric ceramics stand out for their excellent dielectric properties, making them widely used in capacitors. In ferroelectrics, the pyroelectric effect is linked to the electrocaloric effect (ECE), which gives an adiabatic temperature variation (*∆T*) upon the application or removal of an electric field [16]. The two effects can be considered as inverse processes of electrothermal conversion, as for the electro‐mechanical conversion [17]. *∆T* is described by the following Maxwell relation [18]:

(1) $$\Delta T=-\int_{E_{1}}^{E_{2}}\frac{T}{\rho C_{E}}{\left(\frac{\partial D}{\partial T}\right)}_{E}dE$$

Where *E_1_* and *E_2_* are initial and final electric fields, *C_E_* is the specific heat capacity, ρ is the density, *T* is temperature, and *D* is the electric displacement. The thermodynamic definition of the pyroelectric coefficient at constant electric field *p_E_* is:

(2) $$p_{E}={\left.\frac{\partial D}{\partial T}\right)}_{E}={\left.\frac{\partial S}{\partial E}\right)}_{T}\;$$

The second term is a consequence of the Maxwell thermodynamic relations, where *S* is entropy. Therefore, materials with strong electrocaloric effects are also strong pyroelectrics [19]. Equation (2) also implies that a large change in entropy leads to a strong pyroelectric effect. Bai et al. [20] and Liu et al. [21] reported enhanced electrocaloric performance of barium strontium titanate (BST) ceramics, with Ba_0.65_Sr_0.35_TiO_3_ exhibiting the best performance among various compositions [20], indicating that BST is attractive for pyroelectric energy harvesting as well.

Apart from the chemical composition, the geometry and form factor of materials also play a role in determining their properties. Thin films generally exhibit higher breakdown strength than their bulk counterparts due to the reduction of defects and the use of controlled deposition techniques [22, 23]. For instance, PST thin films can withstand up to 1500 kV cm^−1^ electric field and achieve an energy density of 9.1 J cm^−3^ [11]. Nevertheless, thin films are constrained by small thermal mass [24] and difficulty in scaling up due to substrate limitations. Multilayer capacitors (MLCs) offer a practical platform as they combine the scalability of bulk with the high breakdown strength of thin films. In this work, we report barium strontium titanate MLCs (Ba_0.65_Sr_0.35_Ti_0.998_Mn_0.002_O_3_) as a lead‐free candidate for pyroelectric energy harvesting. We performed Olsen cycles under an electric field of 300 kV cm^−1^ from −60°C to 110°C on BST MLCs, and our samples generate 3.7 J cm^−3^ of electricity, comparable to the best lead‐based MLCs illustrated in the work of Lheritier et al. [9, 25]. With a tuned phase transition temperature by substituting Ba^2+^ for Sr^2+^, BST MLCs enable effective energy conversion below 100°C. By employing abundant and low‐cost elements, this approach provides an environmentally friendly and economically viable route toward practical applications.

## 2 Barium Strontium Titanate Multilayer Capacitors

Barium strontium titanate multilayer capacitor samples used in this study are provided by Murata Manufacturing. Figure 1a–c presents the schematic and the scanning electron microscope (SEM) cross‐section of an MLC sample with interdigitated pyroelectric layers and inner electrodes. One sample consists of 11 layers of BST films intercalated with 10 layers of platinum (Pt) electrodes, with the average thickness of 36 µm and 1.6 µm, respectively. Two protective inactive layers of BST films are located at the top and bottom of the stack. The red frame in Figure 1a represents the active part of the sample, which comprises only the BST layers experiencing the electric field and excludes the inactive parts close to both terminal ends. The active volume is 50% of the total volume. Table 1 summarizes the thermal and geometric parameters of BST MLCs. More details are available in the Methods section.

**FIGURE 1:**
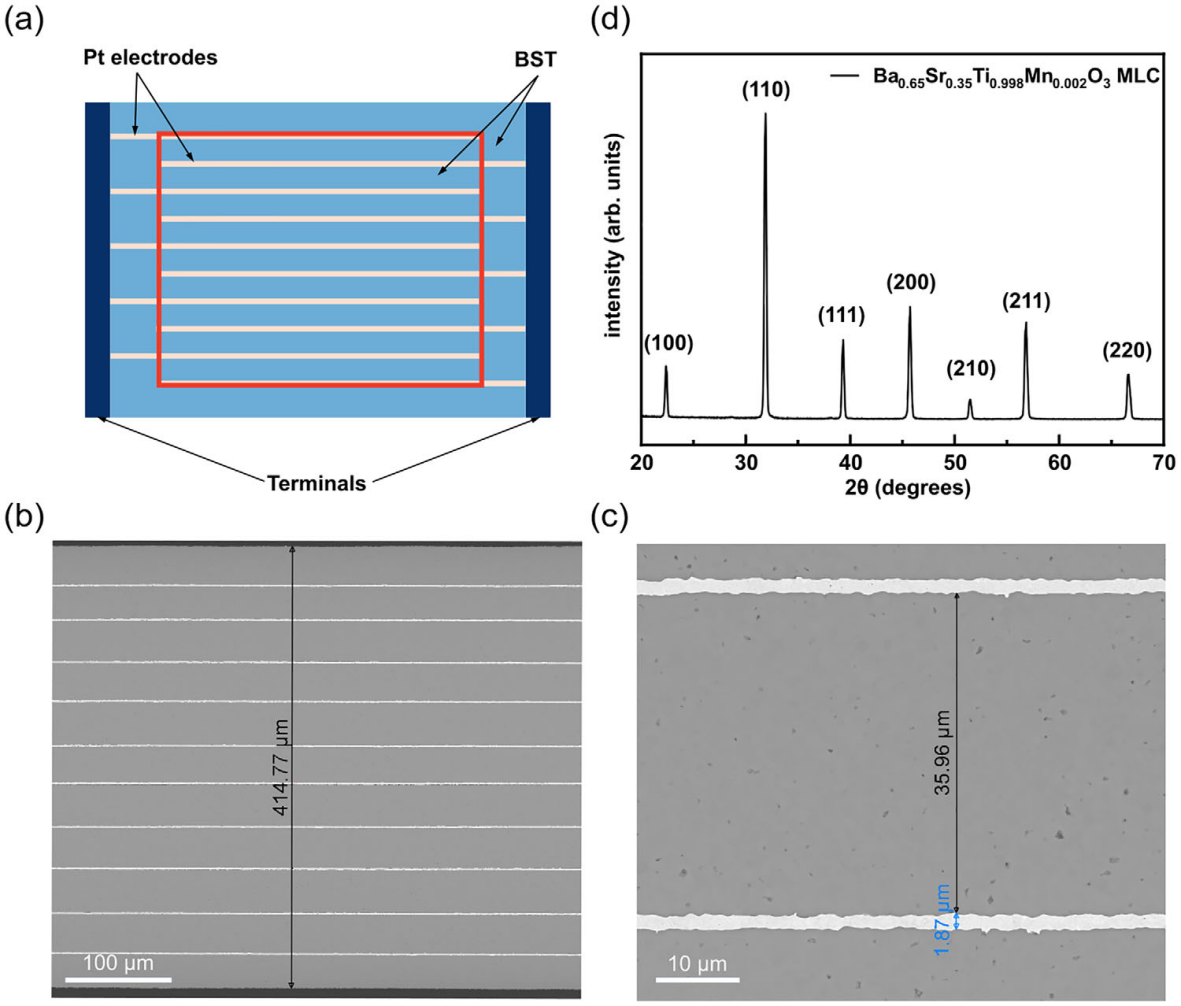
BST MLC structure. (a) Schematic cross‐section of one MLC. Blue layers are the BST ceramics; pink lines indicate inner platinum electrodes; dark regions at both ends represent metal terminals for external contacts; the red frame highlights the active volume of the capacitor. (b, c) Cross‐sectional SEM graph of a single MLC. The average thickness of the entire capacitor (b), one BST layer (c), and one electrode (c) is 415, 36, and 1.6 µm, respectively. (d) XRD pattern of the BST MLC sample recorded at room temperature.

**TABLE 1:** Thermal and geometric parameters of BST MLCs.

Parameter	Value
Number of total layers	11
Number of active layers	9
Number of electrodes	10
Ceramic layer thickness [µm]	36
Electrode thickness [µm]	1.6
Active area [mm^2^]	438.3
Active volume [mm^3^]	15.8
Total volume [mm^3^]	31.5
Specific heat [J K^−1^ g^−1^]	0.47
Density [g cm^−3^]	5.605

Figure 1d shows the X‐ray diffraction (XRD) pattern of one BST MLC measured at room temperature. We observe an expected single‐phase perovskite without secondary phases. Besides, the dominance of the (110) peak shows that there is no preferred orientation, according to the PDF card 04‐023‐4074 in the tetragonal phase [26].

Figure 2a shows the dielectric constant and losses in BST MLC measured at 0.1, 0.5, 1, and 5 kHz during the cooling process. The maximum permittivity was observed at 18°C for all measured frequencies, suggesting the FE‐to‐PE phase transition occurs at 18°C for the given Ba/Sr ratio of 65/35. It is worth noting that while BST transitions from the FE phase to the PE phase, its dielectric loss decreases by ∼90% across all measured frequencies. In addition, BST MLCs show negligible frequency dispersion in their dielectric response. Differential scanning calorimetry (DSC) measurements were performed at zero field to confirm the phase transition, which yields a Curie temperature consistent with that determined by dielectric spectroscopy, as presented in Figure S1. The heat flux profile shows a diffuse peak at the Curie temperature, indicating that BST MLCs undergo a second‐order phase transition. The composition of the investigated BST is adjusted by using 35% of Sr^2+^ and by doping with 0.2% of Mn to tailor the dielectric properties. Besides, substituting Ba^2+^ for Sr^2+^ decreases the phase transition temperature from 120°C to room temperature [27] and thus changes the operating temperatures.

**FIGURE 2:**
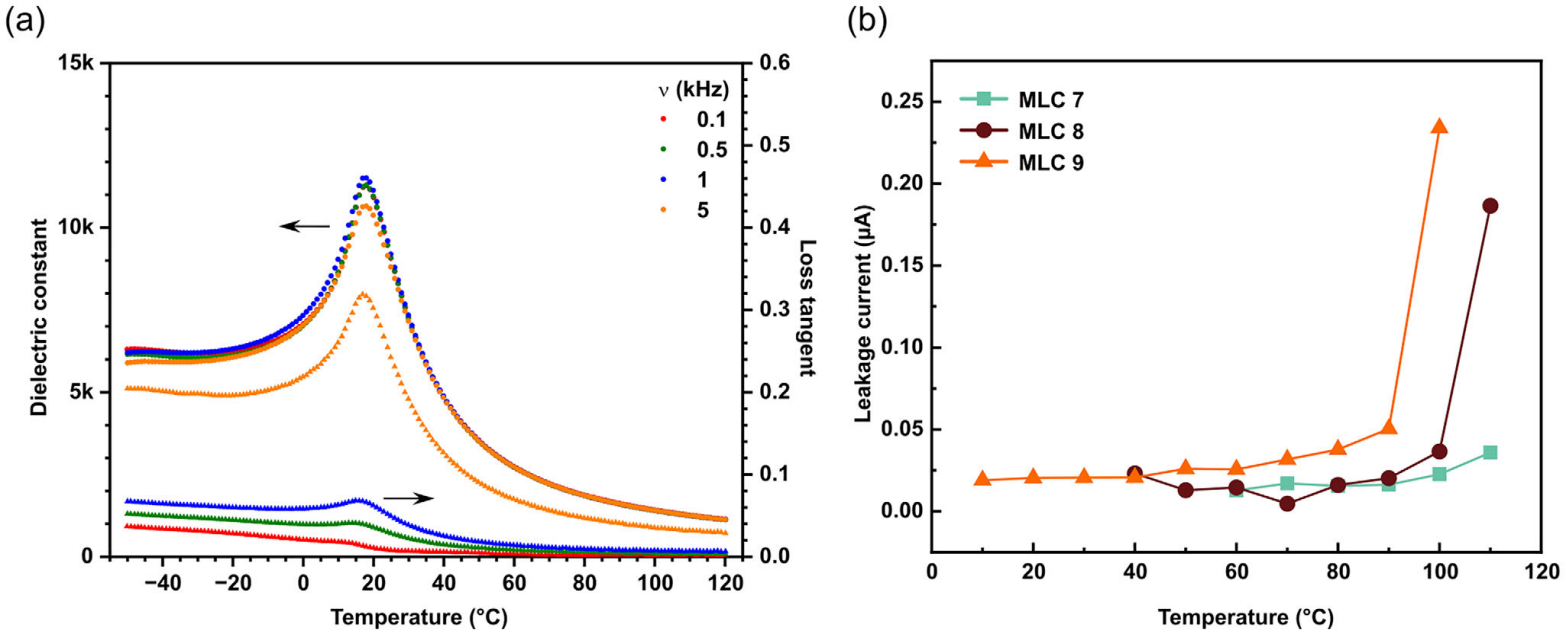
(a) Dielectric measurements in BST MLC showing the FE‐to‐PE phase transition. The temperature‐dependent dielectric constant (left axis) and tangent loss (right axis) were measured at 0.1, 0.5, 1, and 5 kHz during the cooling process. (b) Leakage current vs. temperature of three BST MLCs measured at 306 kV cm^−1^.

Doping with Mn ions has been reported to decrease the concentration of oxygen vacancies in thin films, thereby decreasing leakage current and enhancing the dielectric strength [28, 29, 30]. Figure 2b presents the leakage current of three representative BST MLCs at 306 kV cm^−1^ in various temperature ranges. Below 100°C, the current remains as low as 0.05 µA but rises sharply once the temperature exceeds 100°C. During the preparation of this work, ten BST MLCs were tested, all of which withstand applied electric fields of at least 300 kV cm^−1^ without electrical breakdown.

## 3 Thermodynamic Cycles

In this study, we consider pyroelectric energy harvesting through thermodynamic cycles. Previous studies have explored the feasibility of various thermodynamic cycles in practical applications [31, 32, 33, 34]. The Olsen cycle was selected here as it has been shown to deliver higher energy density [9, 33, 34, 35]. For example, it has been reported that the Olsen cycle typically generates 30% more energy density than the Stirling cycle under the same electric and thermal conditions in *D‐E* loops [9, 33]. Also, fine control of the electric field can be realized throughout the Olsen cycle. By contrast, in Stirling cycles, voltage is amplified during the open‐circuit heating step and may exceed breakdown strength [19].

The Olsen cycle is an electrical analogue of the Ericsson cycle, which is composed of two isothermal and isoelectric field branches. The Olsen cycle can be implemented indirectly and described by *D‐E* loops, as shown in Figure 3a. The sample was first charged isothermally at low temperature *T_low_* (A‐B path), then heated at constant electric field (B‐C path). After reaching the target temperature *T_high_*, it was discharged isothermally (C‐D path) and cooled subsequently to *T_low_* (D‐A path). The pyroelectric energy density (*W*) in such a cycle is the area between the two isothermal *D‐E* loops obtained at two different temperatures:

(3) W=∮EdD

where *E* is the electric field, and *D* is the electric displacement. Therefore, the energy density generated in an Olsen cycle can be extracted from *D‐E* loops at different temperatures, which is referred to as indirect energy harvesting in this study. In the given case in Figure 3a, the energy density indirectly harvested is 2.0 J cm^−3^.

**FIGURE 3:**
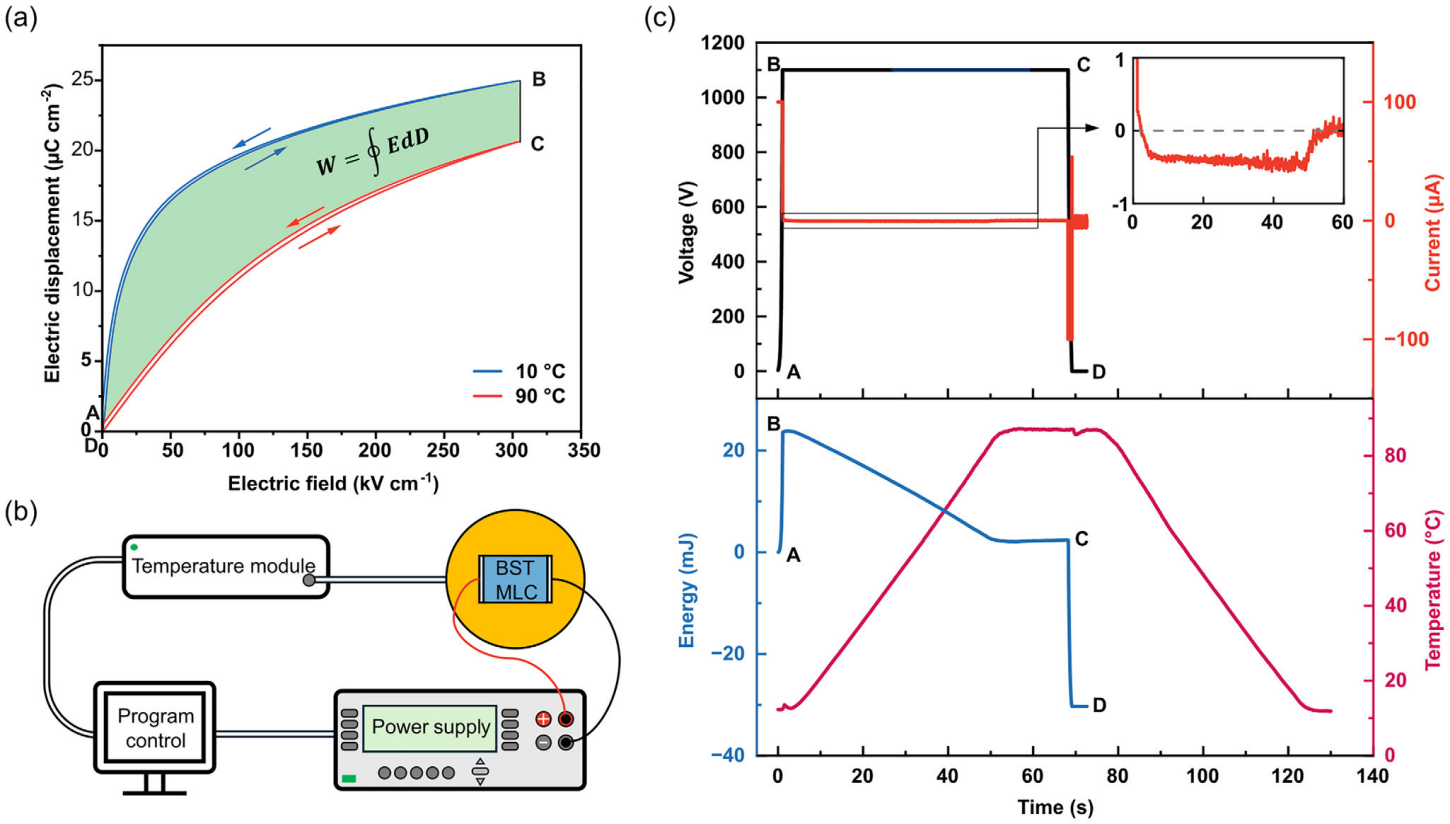
(a) Electric displacement (*D*) – electric field (*E*) schematic of Olsen cycle ABCD between 10°C and 90°C at 306 kV cm^−1^ for pyroelectric energy conversion. For the 10°C loop, the lower half is considered, as it represents the input energy density, whereas for the 90°C loop, the upper half is used to represent the output energy density. The green area corresponds to the net harvested energy density (*W*). (b) Schematic of the experimental setup for direct Olsen cycles. (c) Direct Olsen cycle implemented on a BST MLC. Top: voltage and current of the BST MLC. The inset shows the enlarged view of the current. Bottom: harvested energy and temperature profile of the BST MLC. Letters ABCD refer to the four steps of the Olsen cycle in both panels. A‐B: the sample was charged up to 1100 V (306 kV cm^−1^) at low temperature *T_low_* = 10°C. B‐C: the voltage was kept at 1100 V, and the temperature increased to high temperature *T_high_* = 90°C. C‐D: the sample was discharged at *T_h_*. D‐A: the temperature decreased to *T_low_*.

In practice, the Olsen cycle can also be run directly by controlling temperature and voltage in each step, and the harvestable energy can be measured accurately. Figure 3b shows the schematic of the experimental setup for direct cycles. A Linkam module controlled heating and cooling, and a Keithley 2410 (Tektronix, United States) power supply charged and discharged the sample. A Python script synchronized the two instruments to operate Olsen cycles and record data (time, current, and voltage), which were integrated to obtain the harvested energy (see Section 6).

Figure 3c illustrates a direct Olsen cycle on a BST MLC under the same thermal and electric conditions as shown in Figure 3a. The cycle started from a low temperature *T_low_* = 10°C and an input voltage *V* = 1100 V (corresponding to 306 kV cm^−1^) was applied with a compliance current *I* = 100 µA (A‐B path). The energy input in this step was 24 mJ. Then, the temperature increased to *T_high_* = 90°C (B‐C path). Thanks to the pyroelectric effect, a negative current was generated during the B‐C path as shown in the inset of Figure 3c, leading to the first harvesting step of 22 mJ. Afterward, the voltage decreased to zero with the same compliance current *I* (C‐D path), enabling the collection of 32 mJ in this second harvesting step. Finally, the temperature decreased down to the initial value *T_low_*. In this direct cycle, the total harvested energy was 30 mJ (−24 mJ + 22 mJ + 32 mJ) over an 80 K temperature span, corresponding to an energy density of 1.9 J cm^−3^, which aligned with the result obtained from the indirect cycle (2.0 J cm^−3^).

In the direct measurement, the BST MLC was charged to 1100 V within approximately 1 s, and a similar timescale was observed during discharging. In Figure 3c, two slight fluctuations of temperature are visible at 0 and 70 s due to the electrocaloric effect. The charging and discharging processes cannot be regarded as ideally isothermal in the given case. However, using a very slow charging rate to approach ideal isothermal conditions will not be feasible in reality. Previous studies have evaluated the influence of charging rate on energy density in the direct Olsen cycle and showed that the harvested energy density obtained via an Olsen cycle with 2 s charging time is similar to that of a near‐ideal Olsen cycle, with a difference of only 0.1 J cm^−3^ (∼ 8%) [36]. This indicates that extending the charging and discharging time has a limited impact on the harvested energy. Moreover, the two temperature changes are about 1 K, which is negligible compared with the entire temperature variation. Therefore, the cycle conducted directly can be considered as a practical realization of the Olsen cycle.

Figure 4a presents a series of unipolar *D‐E* loops measured from −60°C to 110°C under an electric field up to 306 kV cm^−1^ at 10 mHz. All *D‐E* loops exhibit slim hysteresis with small remnant polarization (P_r_ < 3 µC cm^−2^) and a nearly zero coercive field. According to Equation (3) and as depicted in Figure 4a, the pyroelectric energy density is calculated over the entire 170 K span and is plotted as the red line in Figure 4b. Besides, direct measurements of the Olsen cycle were independently conducted under the same electric field 306 kV cm^−2^, from −60°C to various final temperatures. The period of one direct Olsen cycle ranges from 82 s to 114 s, depending on the end temperature. Therefore, a frequency of 10 mHz has been selected for *D‐E* loops so that one indirect Olsen cycle lasts 100 s, minimizing the difference in experimental conditions. As shown in Figure 4b, the two methods yield consistent outcomes, with a maximum energy density of 3.7 ± 0.2 J cm^−3^ from −60°C to 110°C. The slight discrepancy above 100°C is analyzed in Section 4. Furthermore, indirect and direct measurements were conducted using eight BST MLCs to validate the reproducibility of the energy harvesting performance (Figure S2). The energy density obtained from both approaches increases with temperature span and shows good agreement in the overlapping temperature range.

**FIGURE 4:**
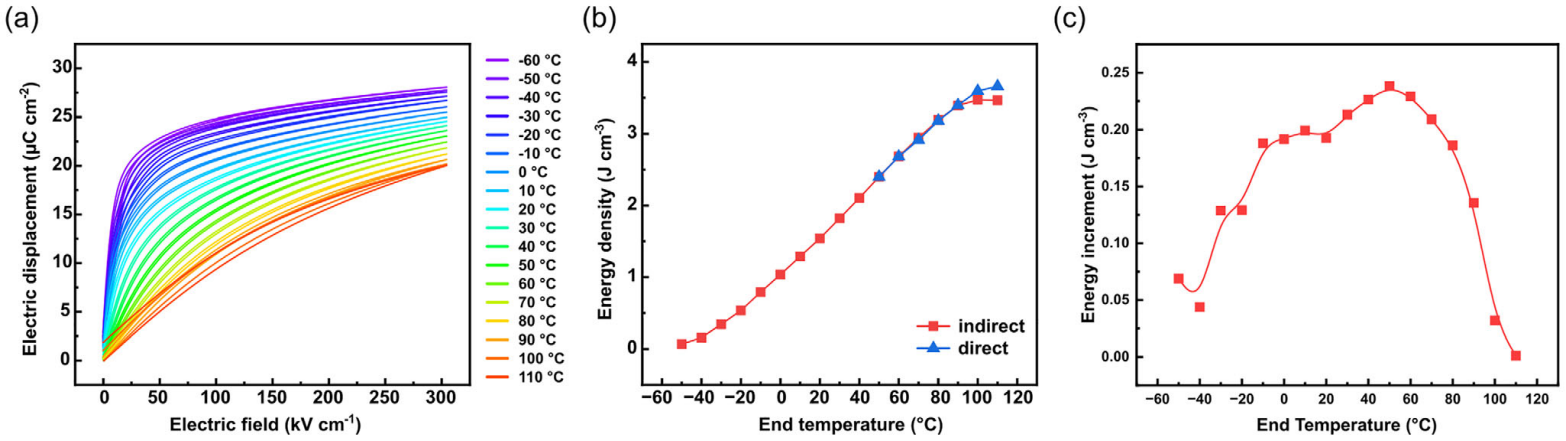
Energy harvesting of a BST MLC. (a) Unipolar *D‐E* loops measured between 0 and 306 kV cm^−1^ at temperatures ranging from −60°C to 110°C and a frequency of 10 mHz. (b) Pyroelectric energy density of a BST MLC from indirect (red) and direct (blue) Olsen cycles at 306 kV cm^−1^ and at various temperature spans. The initial temperature is −60°C in all cases, and the X‐axis corresponds to the high temperature of the cycle. (c) Temperature dependence of energy increment for every 10 K span from −60°C to 110°C at 306 kV cm^−1^. Values extracted from the indirect method.

Additional *D‐E* loop measurements were performed at 10, 20, and 200 mHz. As shown in Figure S3, the harvested energy density curves exhibit similar slopes and amplitudes, suggesting negligible frequency dependence within the investigated range.

To identify the most efficient temperature window for pyroelectric energy harvesting, the energy density is calculated for every 10 K span from −60°C to 110°C based on the *D‐E* loops in Figure 4a. As depicted in Figure 4c, the energy increments follow a concave shape over the 170 K span. The maximum increment of 0.23 ± 0.02 J cm^−3^ is obtained for the cycle from 40°C to 50°C. Between −10°C and 80°C, energy increments are significantly higher than those near the spectrum boundaries. In contrast, energy density increases by only 0.001 J cm^−3^ from 100°C to 110°C, which can be considered negligible.

The net output efficiency *η* and the scaled efficiency *η_r_* (the relative efficiency with respect to Carnot efficiency) were calculated for different initial temperatures (see Supporting Information for details of the calculation). As shown in Figure S4, *η* reaches a maximum of 0.98% (corresponding to *η_r_* = 6.7%) for a −10°C to 90°C cycle, and it remains high over a broad temperature range centered around the FE‐to‐PE phase transition. Notably, *η_r_* peaks at 24.3% in the range from 40°C to 50°C. The results indicate that the phase transition plays an essential role in an efficient Olsen cycle.

## 4 Discussion

Due to the substitution of Sr^2+^ for Ba^2+^, BST MLCs exhibit a diffuse second‐order phase transition behavior rather than a sharp first‐order phase transition in pure BaTiO_3_ [20], leading to a broad temperature range beneficial for pyroelectric energy harvesting. The phase transition temperature depends on the concentration of Ba^2+^ and Sr^2+^ and is also influenced by the applied electric field. Early studies have reported that the Curie temperature (*T_C_*) shifts toward higher values with increasing electric field [37]. Bai et al. [38] similarly observed that the maximum electrocaloric effect moves to higher temperatures under stronger applied fields in BaTiO_3_ multilayer thick films. As the thermodynamic inverse of ECE, pyroelectric performance shows comparable behavior, as illustrated in Figure 4c. The peak of harvested energy increment occurs between 40°C and 50°C, which is higher than the zero‐field *T_C_* of 18°C, indicating a field‐induced shift of the phase transition. Overall, higher energy increments appear between −10°C and 80°C, implying that energy density increases rapidly in the vicinity of the phase transition temperature. Hence, in line with prior studies [9, 35, 39], this result confirms that the pyroelectric energy harvesting is significant near the phase transition due to the strong thermoelectric coupling [31] and the large change in entropy. The optimal operating temperature window lies mainly below 100°C, making these BST MLCs well suited for low‐grade waste heat recovery applications.

In Figure 4a, the *D‐E* loops become slimmer, and the remanent polarization decreases with increasing temperature up to 90°C. This trend suggests that temperature increase destabilizes the ordered ferroelectric state and decreases spontaneous polarization. However, the variation in hysteresis loss is not monotonic. Above 90°C, the loss increases with further temperature rise, which can be attributed to the increase in leakage current. This elevated leakage may also account for the discrepancy between direct and indirect energy densities in Figure 4b. In an indirect Olsen cycle, *D‐E* loops are measured at a constant temperature. At temperatures as high as 100°C, the elevated leakage current persists throughout the entire heating branch (50 s). Hence, during a direct Olsen cycle, significant leakage current occurs only for a few seconds near the end of the heating stage, leading to lower overall energy loss compared with an indirect cycle. Nevertheless, the harvested energy densities obtained from both methods are consistent (Figure S2), which highlights the reproducibility of the Olsen cycle measurements.

The performance of energy conversion via the Olsen cycle in lead‐free and lead‐based pyroelectric materials is provided in Table 2. Among these materials, PST is widely reported as a promising pyroelectric material due to its high pyroelectric coefficient and sharp first‐order FE‐to‐PE phase transition [19, 40]. However, the hazardous constituent Pb in PST is always an environmental concern and thus restricts its application.

**TABLE 2:** Maximum energy density in pyroelectric materials via the Olsen cycle.

Material	Type–thickness [µm]	Zero‐field T_C_ [°C]	T_initial_ [°C]	T_span_ [K]	Electric field [kV cm^−1^]	Energy density [J cm^−3^]	Ref.
BaTiO_3_	Thin film‐ 0.2	120	20	100	25	0.01	[24]
Ba_0.66_Sr_0.34_TiO_3_	Ceramic–200	23	25	40	70	0.36	[41]
Ba_0.72_Sr_0.28_TiO_3_	Ceramic–200	43	25	60	70	0.48	[41]
Pb_0.93_La_0.07_(Zr_0.65_Ti_0.35_)_0.9825_O_3_	Ceramic–190	120	30	170	68	1.01	[42]
Pb_0.92_La_0.08_(Zr_0.65_Ti_0.35_)_0.98_O_3_	Ceramic–290	65	25	135	73	0.89	[43]
PbSc_0.5_Ta_0.5_O_3_	MLC–9 layers of 38 µm each	20	5	175	195	4.4	[9]
Ba_0.65_Sr_0.35_Ti_0.998_Mn_0.002_O_3_	MLC–9 layers of 36 µm each	18	−60	170	306	3.7	This work

To compare fairly, we selected BST MLCs with similar structure and dimensions to PST MLCs to minimize the dimensional effects, as well as a specific composition ratio of Ba^2+^ (65%) and Sr^2+^ (35%) to ensure the equivalent *T_C_* to that of reported PST [25]. When both samples are subjected to direct Olsen cycles over a temperature span of 175 K and under an electric field of 195 kV cm^−1^, the BST MLC delivers an energy density of 2.9 J cm^−3^(Figure S5), accounting for 66% of the benchmark value (4.4 J cm^−3^) reported for PST MLCs [9]. Over a span of 155 K, BST MLCs operated at 306 kV cm^−1^ can withstand twice the electric field applied to PST MLCs (145 kV cm^−1^) due to high dielectric strength. Under these conditions, BST MLCs achieve an energy density of 3.6 J cm^−3^(Figure S6), equivalent to the value of 3.6 J cm^−3^ reported for PST MLCs [25].

In comparison to other lead‐free ceramics, the high applied electric field maximizes the output energy density as well. For example, BST MLCs can sustain an electric field four times higher than that applied to BST bulk ceramics [41]. For the same temperature span of 60 K, BST MLCs harvest 1.6 J cm^−3^, which is three times the energy density obtained from BST bulk ceramics [41]. Besides, compared with BTO thin films and BST ceramics, BST MLCs can work stably over a large variation of temperature, which is important to enlarge the thermodynamic cycle and convert more heat into electricity.

A key contributor to these findings is the high dielectric strength, which enables BST MLCs to reach energy conversion performance comparable to that of PST MLCs and to compensate for their intrinsically lower pyroelectric coefficient. In addition, the FE‐to‐PE phase transition and the broad usable temperature range are also essential for improving the harvested energy. These results support that BST MLCs are promising candidates for pyroelectric energy harvesting, due to their strong heat‐to‐electricity conversion capability, large dielectric strength, practical operation for low‐grade waste heat, and material sustainability.

Finally, we discuss the long‐term operational stability of BST MLCs. In this work, degradation of pyroelectric performance arises primarily from leakage current, which is thermally activated and therefore sensitive to the maximum operating temperature. The temperature dependence of functional lifetime was examined, which shows that lifetime increases rapidly with decreasing temperature (Figure S7). Our analysis shows that the temperature limit to enable cycling 10^6^ times is 60°C (Section S6). Under 500 V (140 kV cm^−1^) with an Olsen cycle from −60°C to 60°C, the harvested energy density reaches approximately 1.5 J cm^−3^. This result positions these BST MLCs preferably toward temperatures below 60°C. However, we intend to improve the propensity of BST MLCs to sustain larger fields at higher temperatures to take full advantage of their harvesting potential revealed in this study.

## 5 Conclusion

This study demonstrates the high performance of lead‐free BST MLCs in non‐linear pyroelectric energy harvesting. A maximum energy density of 3.7 J cm^−3^ is achieved through a direct Olsen cycle over a temperature span of 170 K under an electric field of 306 kV cm^−1^. These BST MLCs notably generate an equivalent energy density over the same temperature span (155 K) as the one reported for PST MLCs when subjected to a direct Olsen cycle, highlighting the essential role of BST MLCs’ high dielectric strength. Besides, the results indicate that greater energy can be harvested near the FE‐to‐PE phase transition and provide insights into selecting optimal temperature windows for energy harvesting. Therefore, BST MLCs are positioned as a sustainable, abundant, and low‐cost alternative to conventional lead‐based materials. We need to improve the capability of such capacitors to withstand large fields at high temperatures to fully benefit from their harvesting potential. Besides, a natural progression of this work is to investigate BST MLCs with varying compositions and Curie temperatures for cascade devices with enhanced energy‐converting efficiency.

## 6 Methods

### 6.1 Samples

MLC 1 was used for the cross‐sectional SEM graph of a single MLC. MLC 2 was used for the XRD pattern at room temperature. MLC 3 was used for dielectric measurements, and MLC 4 was used for zero‐field DSC measurement (Section S1) to identify the FE‐to‐PE phase transition. MLC 5 was used to conduct *D‐E* loops and direct Olsen cycles, from which harvested energy density and efficiency (Section S4) were evaluated. MLC 5 – 8 were used for *D‐E* loops at various frequencies to show the frequency‐independence of pyroelectric energy harvesting performance (Section S3). MLC 6 – 13 were used for verifying reproducibility (Section S2), and leakage current measurements were based on direct cycles of MLC 7 – 9. MLC 14 – 16 were used for accelerated stability testing (Section S6).

### 6.2 BST‐MLC Fabrication

BST MLCs were synthesized and fabricated via solid‐state reaction and tape casting. High‐purity BaCO_3_, SrCO_3_, TiO_2_, and Mn_3_O_4_ were weighed in stoichiometric proportions and mixed. The mixed powder was ball‐milled in distilled water with partially stabilized zirconia balls for 16 h. The dried slurry was calcined at 1150°C for 2 h, then the calcined powder was re‐milled in an organic solvent with binder for 16 h. Green sheets were formed by the doctor blade method. Pt paste was screen‐printed as the inner electrode, and the sheets were stacked, pressed, and cut into green chips. Binder burnout was performed at 500°C for 24 h, followed by sintering at 1350°C for 4 h in air. Finally, silver paste was applied and fired at 750°C to form terminals on the two sides of BST MLCs.

### 6.3 Structure Characterization

The cross‐sectional microstructure of the BST MLCs was examined by a Hitachi SEM SU‐70 (Hitachi High‐Tech Corporation, Japan).

The XRD analysis was carried out on a PANalytical X'Pert PRO MPD instrument (Malvern Panalytical, Netherlands) equipped with a Cu‐K_α_ anode (wavelength: 1.54180 Å) and a PIXcel 3D detector. For the analysis, the detector settings were adjusted to keep any potential signal from fluorescence to a minimum. The measurement was performed in parallel beam configuration, using a Goebel mirror in front of the sealed Cu tube and a parallel plate collimator in front of the detector. The BST MLC was measured as a bulk sample. The scan was conducted in steps of 0.02° over a *2θ* range between 20° and 90° at a scan rate of 0.3°/min at room temperature. Diffraction peaks were indexed using the PDF file 04‐023‐4074 [26].

### 6.4 Dielectric Properties Measurement

The temperature dependence of the dielectric constant and the dielectric loss tangent was measured by a TF Analyzer 2000 (aixACCT Systems, Germany) at 0.1, 0.5, 1, and 5 kHz. Unpoled BST MLCs were heated up from −50°C to 120°C and subsequently cooled back to −50°C, with temperature stabilization every 1°C, under an AC voltage of 0.15 V.

The leakage current was evaluated through direct Olsen cycles. After the sample reached the target high temperature, the applied voltage was maintained, and the pyroelectric current decreased to zero. The current fluctuated around zero until the sample was discharged at the compliance current. The leakage current at each temperature was defined as the average current between reaching the target temperature and discharging. This leakage current led to the energy plateau at the end of heating stage B‐C in Figure 3c. At this moment, pyroelectric current did not contribute, while the leakage current could cause slight energy loss.

### 6.5 Differential Scanning Calorimetry Measurement

Calorimetric measurements were conducted using a commercial differential scanning calorimeter DSC 3+ (METTLER TOLEDO, United Kingdom) on a BST 65/35 MLC. The temperature was swept from −10°C to 50°C at a scanning rate of 5°C/min for both heating and cooling.

### 6.6 Dielectric Hysteresis Loops

Dielectric hysteresis loops were measured with a TF Analyzer 2000 (aixACCT Systems, Germany) with temperature controlled by a Linkam THMS600 high‐temperature stage (Linkam Scientific Instruments, United Kingdom). The specific conditions for each measurement are indicated in the corresponding figure captions in the main text and Supporting Information. Before starting measurements, the Linkam stage was closed and purged with nitrogen to remove the residual air and ensure a dry experimental environment. To avoid the influence of remanent polarization, BST MLCs were depolarized at 120°C between each measurement. *D‐E* loops were performed with samples in a silicon oil medium to isolate them from ambient air and suppress dielectric breakdown. The harvested energy density was estimated indirectly from *D‐E* loops. It was determined by calculating the area enclosed between two *D‐E* loops collected at different temperatures, as illustrated in Figure 3a.

### 6.7 Dedicated Setup for Direct Olsen Cycles

The voltage and current supplied to the BST MLCs were recorded using a Keithley 2410 sourcemeter (Tektronix, United States). A custom Python script was developed to collect voltage and current data as a function of time during charging and discharging of BST MLCs. During charging, a constant current of 0.1 mA was applied for a duration of ∼1 s until the target voltage was reached. The same discharging rate was applied during discharging.

The net energy exchange was then determined by integrating the instantaneous voltage *V(t)* and current *I(t)* over one cycle:

(4) $$W=\int_{0}^{t_{0}}I\left(t\right)V\left(t\right)dt$$

where *t_0_* is the duration of one cycle. In the energy profile, positive values correspond to energy input supplied to the BST MLCs, whereas negative values represent the net harvested energy.

## Author Contributions

**Junning Li**: investigation, data curation, writing – review and editing. **Moiz Khalil**: writing – review and editing. **Ashwath Aravindhan**: writing – review and editing. **Uros Prah**: investigation, writing – review and editing, data curation, visualization. **Torsten Granzow**: validation, writing – review and editing, investigation. **Emmanuel Defay**: conceptualization, methodology, supervision, writing – review and editing, project administration, funding acquisition. **Youri Nouchokgwe**: investigation, writing – review and editing, formal analysis, visualization. **Sakyo Hirose**: writing – review and editing, resources. **Veronika Kovacova**: writing – review and editing, supervision, methodology, conceptualization, project administration. **Fan Ni**: writing – original draft, visualization, validation, formal analysis, investigation, data curation, writing – review and editing. **Yves Fleming**: writing – review and editing, investigation, formal analysis.

## Funding

This project has received funding from the European Research Council (ERC) under the European Union's Horizon Europe programme (Grant agreement No. 101141445 ELEC_FROM_HEAT).

## Conflicts of Interest

The authors declare no conflicts of interest.

## Supporting information




**Supporting file**: advs77106‐sup‐0001‐SuppMat.docx

## Data Availability

The data that supports the findings of this study are available in the supplementary material of this article.
