# Supplementary material for: Heat‐to‐Electricity Conversion Using Barium Strontium Titanate Multilayer Capacitors
Source: Adv Sci (Weinh). 2026 Aug 21;13(62):e77106. doi: 10.1002/advs.77106 (PMC13495953; doi:10.1002/advs.77106)
Supplement: Supplementary file 1 [file ADVS-13-e77106-s001.docx]

# Supplementary Material

**Heat-to-Electricity Conversion Using Barium Strontium Titanate Multilayer Capacitors**

*Fan Ni ^a, b^, Junning Li ^a, c^, Uros Prah ^a, d^, Youri Nouchokgwe ^a^, Yves Fleming ^a^, Ashwath Aravindhan ^a^, Moiz Khalil ^a, b^, Torsten Granzow ^a^, Sakyo Hirose ^e^, Veronika Kovacova ^a^, and Emmanuel Defay ^a, b,^ **

^a^ Luxembourg Institute of Science and Technology (LIST), Maison des Materiaux, 4362 Esch-sur-Alzette, Luxembourg

^b^ Department of Physics and Materials Science, University of Luxembourg, Maison des Materiaux, 4362 Esch-sur-Alzette, Luxembourg

^c^ Present address: Hunan University, Changsha, China

^d^ Present address: Department of Materials and Earth Sciences, Technical University of Darmstadt, Darmstadt, Germany

^e^ Murata Manufacturing Co., Nagaokakyo, Kyoto 617-8555, Japan

* Corresponding author

Email: emmanuel.defay@list.lu

## Contents

Section 1. Differential Scanning Calorimetry (DSC) Measurement

Section 2. Reproducibility of Pyroelectric Energy Harvesting

Section 3. Frequency Independence of Pyroelectric Energy Harvesting

Section 4. Net Output Efficiency

Section 5. Comparison with PST MLCs

Section 6. Accelerated Stability Testing

### Section 1. Differential Scanning Calorimetry (DSC) Measurement

DSC measurements were performed on a BST 65/35 MLC at a scanning rate of 5 °C/min for both heating and cooling. The heat flow curves display well-defined endothermic and exothermic peaks. The diffuse peaks indicate the second-order phase transition in Ba_0.65_Sr_0.35_TiO_3_. On heating, the FE-to-PE transition occurs at 17 °C, while on cooling the reverse transition is observed at 16 °C, suggesting a thermal hysteresis of only 1 °C. The latent heat associated with the phase transition was quantified by integrating the area under each peak, which is 202 J kg^-1^.

**Figure S1:** The heat flux of BST 65/35 MLC as a function of temperature.

### Section 2. Reproducibility of Pyroelectric Energy Harvesting

Additional indirect and direct Olsen cycle measurements at 306 kV cm^-1^ were performed over various temperature spans with eight BST MLCs.

**Figure S2:** (a) Pyroelectric energy density (mean ± SD, n ≥ 4) of BST MLCs from indirect (red) and direct (blue) Olsen cycles at 306 kV cm^-1^ and at various temperature spans. (b) Temperature-dependence of energy increment (mean ± SD, n ≥ 4) for every 10 K span from -40 °C to 110 °C at 306 kV cm^-1^. Values extracted from the indirect method.

### Section 3. Frequency Independence of Pyroelectric Energy Harvesting

Three MLCs were used to perform *D-E* loop measurements at different frequencies. For the presented 150 K temperature span, the mean values of harvested energy density at 10, 20, and 200 mHz are 3.5, 3.2, and 3.3 J cm^-3^, respectively. As shown in Figure S3, three curves remain at similar slopes and amplitudes, exhibiting negligible frequency dependence.

**Figure S3:** Pyroelectric energy density measured at 306 kV cm^-1^ between -40 °C to 110 °C at 10, 20, and 200 mHz.

### Section 4. Net Output Efficiency

The net output efficiency *η* is the ratio of net output electric work *W* over input heat *Q_in_*:

$\eta=\frac{W}{Q_{in}}$(1)

The input heat can be described as

$Q_{in}=\rho C_{p}(\Delta T+\Delta T_{EC})$(2)

where *ρ* is the density and *C_p_* is the specific heat of BST 65/35, respectively; *ΔT* is the temperature increase in Olsen cycle and *ΔT_EC_* is the temperature variation caused by electrocaloric effect when the electric field is removed. A detailed description of the calculation and approximation of input heat can be found in the works of Lheritier et al.^[1]^ and Nouchokgwe et al.^[2]^ .

Carnot efficiency is the ideal efficiency of energy conversion between two temperatures, which is defined as

$\eta_{Carnot}= \frac{\Delta T}{T_{hot}}$ (3)

The scaled efficiency *η_r_* is the ratio of net output efficiency over Carnot efficiency:

$\eta_{r}= \frac{\eta}{\eta_{Carnot}}= \frac{WT_{hot}}{\rho C_{p}(\Delta T+\Delta T_{EC})\Delta T}$ (4)

For BST 65/35, the density is 5.605 g cm^-3^ (according to the sample supplier), and the specific heat is 0.47 J g^-1^ K^-1^ (measured in our lab by differential scanning calorimetry). The average *ΔT_EC_* at 306 KV cm^-1^ and 25 °C is ~2 K^[3]^. The output electric work is extracted from the *D-E* loops shown in Figure 4 of the main text.

**Figure S4:** The pyroelectric efficiency *η* (a) and the scaled efficiency *η_r_* (b) of a BST 65/35 MLC as a function of temperature span *ΔT*. Values are calculated based on energy density results at 306 kV cm^-3^ in Figure 4 of main text and different initial temperatures *T_i_*.

### Section 5. Comparison with PST MLCs

**Figure S5:** Direct Olsen cycle implemented on one BST MLC under 700 V (195 kV cm^-1^) from -70 °C to 105 °C. The harvested energy is 45.2 mJ, corresponding to 2.9 J cm^-3^.

**Figure S6:** Direct Olsen cycle implemented on one BST MLC under 1100 V (306 kV cm^-1^) from -60 °C to 95 °C. The harvested energy is 56.6 mJ, corresponding to 3.6 J cm^-3^.

## **Section 6. Accelerated Stability Testing**

In direct Olsen cycles, degradation of pyroelectric performance arises primarily from leakage current. This degradation becomes most pronounced near the near the end of the heating stage, where the sample is simultaneously exposed to target high temperature and sustained high electric field for ~5.7 s per cycle. In our measurements, an average leakage current of 0.32 μA leads to approximately 10% reduction of harvested energy density at 120 °C, which could be considered as indicative of functional degradation in real scenarios. To assess this issue, we conducted accelerated stability testing under elevated temperatures and DC bias to evaluate the lifetime of BST MLCs and the evolution of their pyroelectric energy harvesting performance under long-term thermal-electrical loading.

As presented in Figure S7(a), the sample was subjected to a DC voltage of 500 V (140 kV cm^-1^) at 90 °C. Because of equipment constraints, one measurement was limited to 16000 s, and three runs were carried out continuously. After a total duration of 48000 s, corresponding to ~10^4^ Olsen cycles, the leakage current was 0.29 μA, approaching the threshold of functional performance degradation. The same voltage was applied to the sample at 120 °C and 170 °C. At 170 °C, the leakage current exceeded the defined threshold of 0.32 μA from the very beginning of the measurement, after 0.1 cycle.

As shown in our experiments, charge migration is thermally activated. To extend this assessment to practical operating conditions, the temperature dependence of functional lifetime (black points) was plotted in log scale with a linear fit (red line) as shown in Figure S7(b). This graph shows that if one wants to reach 10^6^ cycles (blue point), the temperature should not surpass 60 °C.

In addition, under 500 V (140 kV cm^-1^) with an Olsen cycle from −60 °C to 60 °C, the harvested energy density reaches approximately 1.5 J cm^-3^. Combined with the lifetime of ~10^6^ cycles, this demonstrates that BST MLCs can reliably deliver ~1.5 J cm^-3^ per cycle over their functional lifetime, confirming their potential for practical pyroelectric energy harvesting applications.

**Figure S7:** (a) Continuous measurements of current as a function of time under 500 V of DC voltage and at 90 °C. (b) The functional lifetime measured at 170 °C, 120 °C , and 90 °C (black points), and a linear fit extrapolated to 60 °C (blue point).

## **Reference**

1. B. Nair, T. Usui, S. Crossley, et al., “Large Electrocaloric Effects in Oxide Multilayer Capacitors over a Wide Temperature Range,” *Nature* *575*, no. 7783 (2019): 468–472, https://doi.org/10.1038/s41586-019-1634-0.

2. Y. Nouchokgwe, P. Lheritier, C.-H. Hong, et al., “Giant Electrocaloric Materials Energy Efficiency in Highly Ordered Lead Scandium Tantalate,” *Nature Communications* *12*, no. 1 (2021): 3298, https://doi.org/10.1038/s41467-021-23354-y.

3. S. Sadhukhan, N. Zeggai, B. Dkhil, S. Hirose, M. Lobue, and M. Almanza, “Electrocaloric Cooling Efficiency: Comparative Insights on P(VDF-TrFE-CFE) Polymer and BSTM Ceramics,” *Ceramics International* (2026), https://doi.org/10.1016/j.ceramint.2026.02.344.
